# A multi-locus species phylogeny of African forest duikers in the subfamily *Cephalophinae*: evidence for a recent radiation in the Pleistocene

**DOI:** 10.1186/1471-2148-12-120

**Published:** 2012-07-23

**Authors:** Anne R Johnston, Nicola M Anthony

**Affiliations:** 1 University of New Orleans, New Orleans, LA, 70148, USA

**Keywords:** Duiker, Pleistocene refuge hypothesis, Speciation, Molecular clock, Partition, Species tree

## Abstract

**Background:**

Duikers in the subfamily Cephalophinae are a group of tropical forest mammals believed to have first originated during the late Miocene. However, knowledge of phylogenetic relationships, pattern and timing of their subsequent radiation is poorly understood. Here we present the first multi-locus phylogeny of this threatened group of tropical artiodactyls and use a Bayesian uncorrelated molecular clock to estimate divergence times.

**Results:**

A total of 4152 bp of sequence data was obtained from two mitochondrial genes and four nuclear introns. Phylogenies were estimated using maximum parsimony, maximum likelihood, and Bayesian analysis of concatenated mitochondrial, nuclear and combined datasets. A relaxed molecular clock with two fossil calibration points was used to estimate divergence times. The first was based on the age of the split between the two oldest subfamilies within the Bovidae whereas the second was based on the earliest known fossil appearance of the Cephalophinae and molecular divergence time estimates for the oldest lineages within this group. Findings indicate strong support for four major lineages within the subfamily, all of which date to the late Miocene/early Pliocene. The first of these to diverge was the dwarf duiker genus *Philantomba*, followed by the giant, eastern and western red duiker lineages, all within the genus *Cephalophus*. While these results uphold the recognition of *Philantomba*, they do not support the monotypic savanna-specialist genus *Sylvicapra*, which as sister to the giant duikers leaves *Cephalophus* paraphyletic. BEAST analyses indicate that most sister species pairs originated during the Pleistocene, suggesting that repeated glacial cycling may have played an important role in the recent diversification of this group. Furthermore, several red duiker sister species pairs appear to be either paraphyletic (*C.callipygus*/*C. ogilbyi* and *C. harveyi*/*C. natalensis*) or exhibit evidence of mitochondrial admixture (*C. nigrifrons* and *C. rufilatus*), consistent with their recent divergence and/or possible hybridization with each other.

**Conclusions:**

Molecular phylogenetic analyses suggest that Pleistocene-era climatic oscillations have played an important role in the speciation of this largely forest-dwelling group. Our results also reveal the most well supported species phylogeny for the subfamily to date, but also highlight several areas of inconsistency between our current understanding of duiker taxonomy and the evolutionary relationships depicted here. These findings may therefore prove particularly relevant to future conservation efforts, given that many species are presently regulated under the Convention for Trade in Endangered Species.

## Background

The Equatorial forests of central Africa are one of the most biologically diverse regions in the world. Understanding the mechanisms that gave rise to such extraordinary tropical diversity remains a subject of intense interest to evolutionary and conservation biologists [1,2]. Critical to this debate has been the role that Pleistocene climate change has played in tropical vertebrate speciation. The periodic fluctuations in climatic conditions that resulted from Earth’s orbital shifts are well known to have affected the range dynamics of many temperate taxa [3]. However, the extent to which these fluctuations impacted the distribution and diversification of tropical forest taxa has been a subject of intense debate. Proponents of Pleistocene refuge theory (e.g. [4]) have argued that the drier, cooler temperatures experienced during successive glacial maxima led to the repeated fragmentation of formerly contiguous forests, which in turn led to allopatric speciation of forest-associated taxa. Although there is currently little evidence to suggest that tropical forest refugia played a major role in the Amazon [5-7], support for their role as drivers of evolutionary diversification in tropical Africa is much greater (e.g. [8-12]). However, the majority of studies to date suggest that these effects are most evident at the population level [8,11-16] but see [17] with most species divergence times pre-dating the Pleistocene [18-20].

The duikers in the subfamily Cephalophinae (family Bovidae) constitute an ideal group for testing the role of Pleistocene refugia in tropical vertebrate speciation because of their recent origin in the Late Miocene and subsequent rapid radiation [21,22]. Currently, three duiker genera are recognized: (a) the recently derived, species-rich, forest dwelling *Cephalophus* (b) the dwarf *Philantomba* and (c) the monotypic savanna specialist *Sylvicapra*. Jansen van Vuuren and Robinson [22] have further sub-divided *Cephalophus* into three major mitochondrial lineages comprising the giant duikers (*C. silvicultor, C. spadix, C. dorsalis,* and *C. jentinki*), east African red duikers (*C. leucogaster, C. rufilatus, C. nigrifrons, C. natalensis, C. rubidus,* and *C. harveyi*) and west African red duikers (*C. callipygus, C. weynsi, C. ogilbyi,* and *C. niger*). To date, support for these lineages has been based solely on single mitochondrial gene genealogies that may not accurately reflect the evolutionary history of this group. Furthermore, the position of the two remaining taxa, *C. adersi* and *C. zebra*, remains unresolved and appears to be highly labile between studies [22-24].

The goal of the present study is therefore to estimate a well-supported species tree for duikers using a combination of mitochondrial genes and nuclear introns previously shown to be highly informative in resolving relationships between other closely-related African bovids [25,26]. We then use this tree to re-evaluate the evolutionary relationships of major lineages within this group and test the hypothesis that speciation of many duikers occurred during the Pleistocene epoch using a fossil calibrated relaxed molecular clock [27].

## Results

The final aligned data matrix contained four unlinked nuclear DNA regions and two mitochondrial DNA regions for a total of 4152 characters, of which 1172 were from mitochondrial and 2980 were from nuclear loci (Table 1). Results from the pair-wise ILD test between the two mitochondrial partitions fail to reject the null hypothesis that both loci are congruent. As expected, the mitochondrial partition contained a greater proportion of variable sites (38%) relative to the nuclear matrix (15%). The mitochondrial partition contained 368 parsimony informative characters (31%) while the nuclear partition contained 208 parsimony informative characters (8%). The consistency index (CI) and retention index (RI) values in the mitochondrial partition (CI: 0.417, RI: 0.621) are lower than those of the nuclear matrix (CI: 0.823, RI: 0.846), indicating higher levels of homoplasy in the mitochondrial dataset.

**Table 1:** Sequence variability

**Marker**	**Aligned length**	**Average unaligned length (Range)**	**Variable sites (%)**	**PIC (%)**	**CI, RI**	**BIC model**
MGF	759	696 (617–727)	137 (18)	64 (8)	0.85, 0.871	HKY + G
PKRCl	552	535 (469–548)	88 (16)	54 (10)	0.892, 0.884	HKY + G
SPTBN1	894	843 (508–871)	130 (15)	64 (7)	0.873, 0.912	HKY + I
THY	775	694 (372–770)	104 (13)	46 (6)	0.891, 0.912	HKY + G
Mitochondrial	1172	1117 (514–1172)	447 (38)	368 (31)	0.417, 0.621	HKY + G + I
Nuclear	2980	2770 (2158–2895)	459 (15)	208 (7)	0.823, 0.846	HKY + G

Patterns of sequence variability among individual mitochondrial and nuclear markers, combined mitochondrial genes, combined nuclear loci, and combined mitochondrial and nuclear loci. These data comprise the number and percentage of variable sites, the number and percentage of parsimony informative characters (PIC), the consistency (CI) and retention (RI) indices, and the suggested model of nucleotide substitution, as selected under the Bayesian information criterion (BIC). GTR, HKY, G and I represent the General Time Reversible model, the Hasegawa-Kishino-Yano model, the alpha shape parameter describing gamma distributed rate variation, and a proportion of invariant sites, respectively.

**Figure 1:**
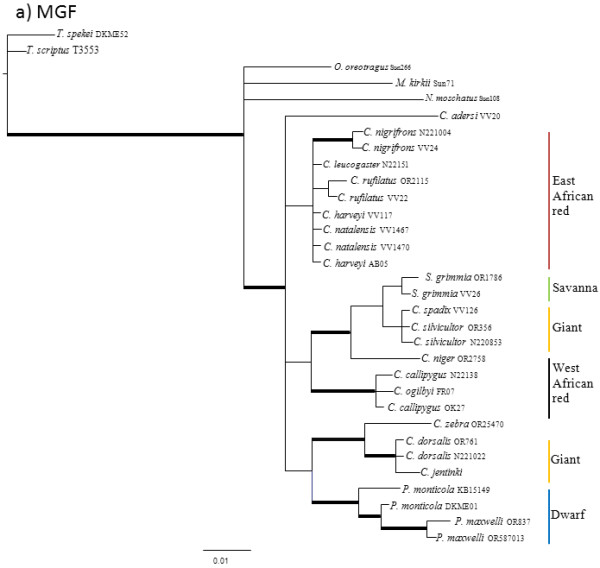
**MGF gene genealogy.** Majority-rule consensus tree showing the Bayesian estimate of nuclear gene trees for MGF. Thickened branches indicate nodal support by both BA posterior probability (PP) values ≥ 0.95 and ML bootstrap support (BS) ≥ 75. Additional file 1: Table S1 lists support values by node for this phylogeny.

**Figure 2:**
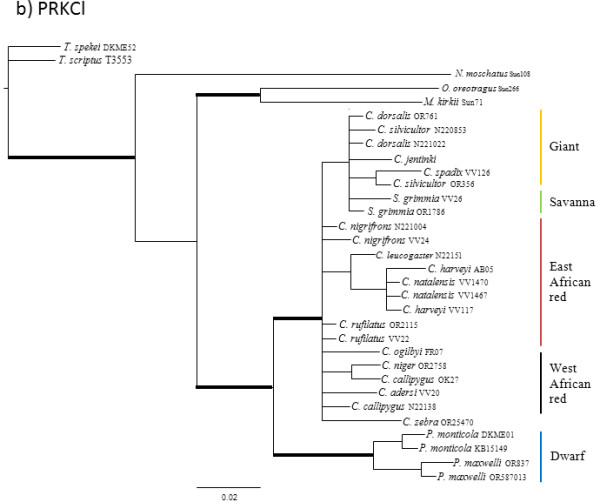
**PRKCl gene genealogy.** Majority-rule consensus tree showing the Bayesian estimate of nuclear gene trees for PRKCl. Thickened branches indicate nodal support by both BA posterior probability (PP) values ≥ 0.95 and ML bootstrap support (BS) ≥ 75. Additional file 1: Table S1 lists support values by node for this phylogeny.

**Figure 3:**
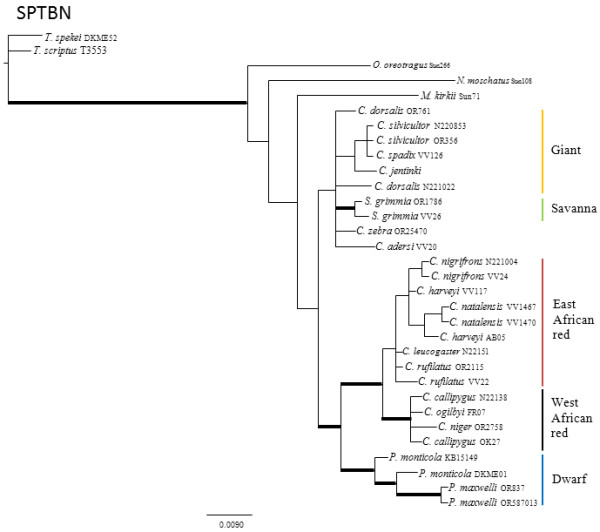
**SPTBN gene genealogy.** Majority-rule consensus tree showing the Bayesian estimate of nuclear gene trees for SPTBN. Thickened branches indicate nodal support by both BA posterior probability (PP) values ≥ 0.95 and ML bootstrap support (BS) ≥ 75. Additional file 1: Table S1 lists support values by node for this phylogeny.

**Figure 4:**
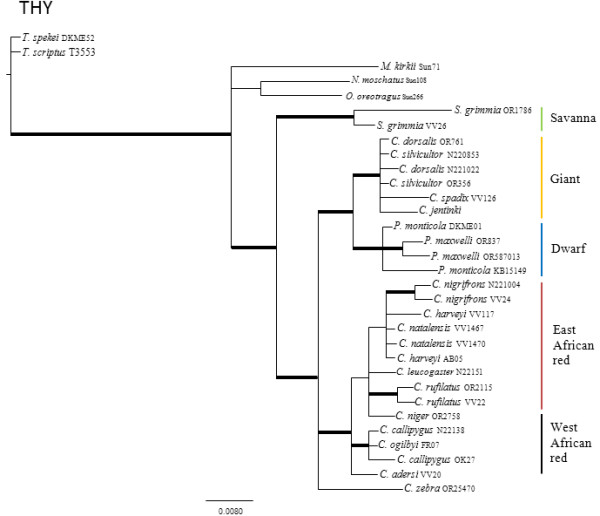
**THY gene genealogy.** Majority-rule consensus tree showing the Bayesian estimate of nuclear gene trees for THY. Thickened branches indicate nodal support by both BA posterior probability (PP) values ≥ 0.95 and ML bootstrap support (BS) ≥ 75. Additional file 1: Table S1 lists support values by node for this phylogeny.

The results of the pair-wise ILD tests between nuclear loci reject the null hypotheses that the four nuclear genes are congruent with one another (p < 0.10). Individual nuclear gene genealogies estimated using Maximum Parsimony (MP), Maximum Likelihood (ML) and Bayesian Analysis (BA) methods recovered different topologies (Figures 1, 2, 3 and 4; Additional file 1 for node support values) and these differences were evident regardless of method. Given that there was generally little support for most of the nodes within the individual nuclear gene trees, many of these differences between gene genealogies likely represent soft polytomies. However, there is also significant support for some of these differences. For example, the MGF genealogy supports two clades of giant duikers (MP bootstrap = 63% and 94%, ML bootstrap = 75% and 92%, BA posterior probability = 0.99 and 1.00) while the THY genealogy supports only one clade (MP = 84, ML = 85, BA = 0.99). The THY genealogy also supports the placement of *S. grimmia* as sister to the remaining Cephalophinae (MP = 90, ML = 91, BA = 1.00) with *Philantomba* sister to the giant duikers (MP = 89, ML = 93, BA = 1.00), unlike the PRKCl genealogy, which supports *Philantomba* as sister to the remaining Cephalophinae (MP = 83, ML = 81, BA = 1.00) or the SPTBN genealogy, which supports *Philantomba* as sister to only the red duiker clades (MP = 73, ML = 76, BA = 1.00).

All four genealogies support the monophyly of *Philantomba* (MGF: MP = 95%, ML = 95%, BA =1.0; PRKCl: MP = 96%, ML = 100%, BA = 1.0; STBN1: MP = 97%, ML = 97%, BA = 1.0; THY: MP = 83%, ML = 87%, BA = 1.0)*. Sylvicapra* was sister to some or all of the giant duikers in three of the four nuclear trees (MGF: MP = 66%, BA = 0.52%; PRKCl: ML = 54%, BA = 0.61; STNB1: MP = 60%, ML = 56%, BA = 0.97) but this relationship lacked significant support. Within *Cephalophus*, support was generally weak or lacking for the giant, east and west African red duiker lineages described by van Vuuren and Robinson [22] although the STBN1 genealogy supported the monophyly of the west African red duiker lineage (MP = 85%, ML = 88%, BA = 1.0) and the THY genealogy recovered the giant duiker lineage (MP = 84%, ML = 85%, BA = 0.99). The position of *C. adersi* and *C. zebra* varied across genealogies and remained unresolved or weakly supported, with the exception of the MGF genealogy which supported *C. zebra* as sister to the *C. jentinki/C. dorsalis* clade (MP = 94%, ML = 92%, BA = 1.0) and the THY genealogy which supported *C. adersi* as sister to the east and west African red duikers (MP = 82%, ML = 80%, BA = 1.0).

**Figure 5:**
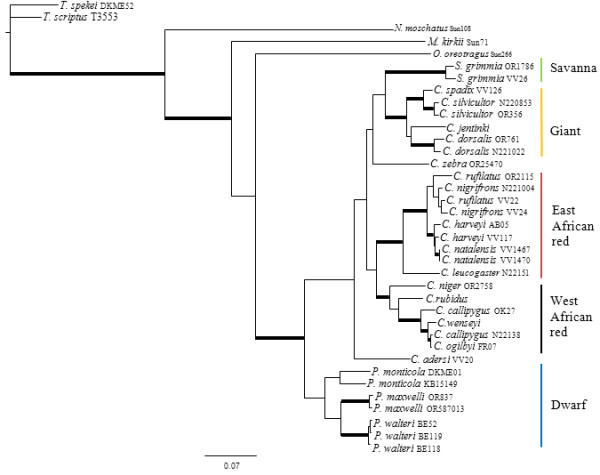
**Mitochondrial gene genealogy.** Majority-rule consensus tree showing the Bayesian estimate of the complete mitochondrial dataset. Thickened branches indicate nodal support by both BA posterior probability (PP) values ≥ 0.95 and ML bootstrap support (BS) ≥ 75. Additional file 1: Table S1 lists support values by node for this phylogeny.

As results from ILD and SH tests suggested that the mitochondrial and nuclear topologies are incongruent (p ≤ 0.006), both datasets were first analysed separately and then combined. The mitochondrial tree of all species within Cephalophinae (Figure 5) shows weak support for the monophyly of *Philantomba* (MP = 88%, ML = 53%, BA = 0.76), but has strong support for the sister placement of these taxa relative to all other Cephalophinae (MP = 68%, ML = 91%, BA = 1.0). *Sylvicapra* is sister to the giant duikers, although this node has weak support (MP = 32%, ML = 58%, BA = 0.84). Within *Cephalophus*, there is strong support for the monophyly of the giant duikers (MP = 98%, ML = 97%, BA = 1.0%), the east African red duikers (MP = 93%, ML = 98%, BA = 1.0), and the west African red duikers (MP = 87%, ML = 83%, BA = 0.99), but weak support for their placement relative to one another. The position of *C. zebra* and *C. adersi* is unresolved. There is also weak support for the paraphyly of *C. rufilatus* relative to *C. nigrifrons* (MP = 59%, ML = 44%, BA = 0.87) and strong support for the paraphyly of *C. callipygus* relative to *C. ogilbyi and C. weynsi* (MP = 98%, ML = 97, BA = 1.0).

In nuclear concatenated matrices, the harmonic mean of the log likelihood of the partitioned combined mitochondrial and nuclear Bayesian analysis (hm_2_) was equal to −15370.43 compared to the log likelihood for the unpartitioned analysis (hm_1_) of −15851.64, giving a value of 2 ln BF = −962.42 and providing strong evidence against a partitioned model. Alternatively, when the mitochondrial data are excluded from analyses, Bayes Factor analysis found strong evidence for the partitioned model (hm_2_ = −8088.25, hm_1_ = −7976.32, 2 ln BF = 223.86).

**Figure 6:**
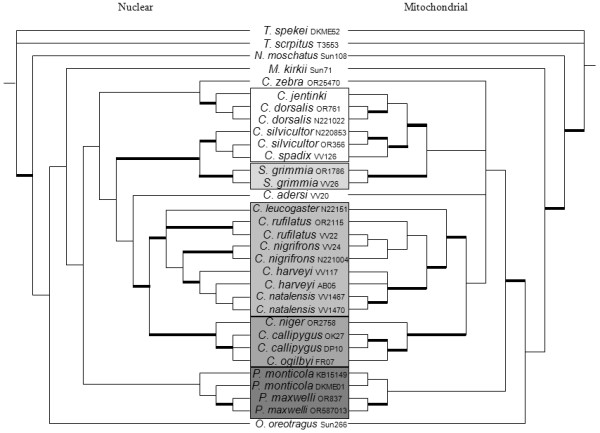
**Combined nuclear tree.** Majority-rule consensus cladogram showing the Bayesian estimate of the species tree from nuclear concatenated (left) and mitochondrial datasets. Thickened branches nodal indicate support by both BA posterior probability (PP) values ≥ 0.95 and ML bootstrap support (BS) ≥ 75. Additional file 1: Table S1 lists support values by node for this phylogeny. Boxes show major lineages on a grey scale, starting with the giant duikers in white, then the savannah duiker, the east African red duikers, the west African red duikers, and the dwarf duikers in darkest grey.

The concatenated nuclear tree (Figure 6) shows strong support for the monophyly of *Philantomba* (MP bootstrap = 100%, ML un-partitioned/partitioned bootstrap = 100/100, BA un-partitioned/partitioned posterior probability = 1.0/1.0). However, the sister position of this genus relative to the other duikers is not supported. Nuclear analyses also support a sister relationship between *Sylvicapra* and the *C. silvicultor/C. spadix* group (MP = 75%, ML = 66/75%, BA = 1.0/0.98), making both the genus *Cephalophus* and the giant duiker lineage paraphyletic. There is also support for the monophyly of the east African red duiker lineage (MP = 91%, ML = 91/98%, BA = 0.99/1.0), the west African red duiker lineage (MP = 67%, ML = 75/86%, BA = 1.0/1.0), and a sister relationship between these two red African duiker lineages (MP = 95%, ML = 100%/100%, BA = 1.0/0.82). *Cephalophus adersi* is sister to both the east and west African red duikers (MP = 60%, ML = 93/91%, BA = 0.93/0.74) and *C. zebra* is sister to the *C. jentinki/C. dorsalis* group (MP = 77%, ML = 59/70%, BA = 1.0/0.98). Unlike the mitochondrial tree, *C. rufilatus* and *C. nigrifrons* form reciprocally monophyletic clades (MP = 86%, ML = 82/95%, BA = 1.0/1.0 and MP = 99%, ML = 99/100%, BA = 1.00, respectively) in the nuclear tree. However, *C. harveyi* is paraphyletic with respect to *C. natalensis*, as is *P. monticola* to *P. maxwelli*. Finally, *C. callipygus and C. ogilbyi* form an unresolved polytomy.

**Figure 7:**
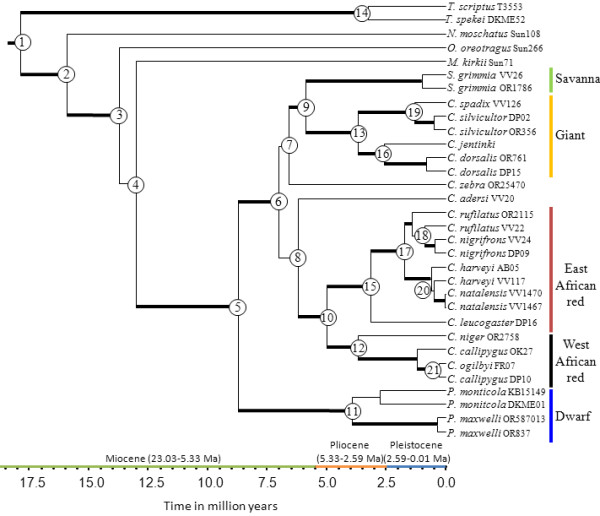
**Species tree.** Chronogram illustrating species relationships and divergence times based on Bayesian analysis of the total evidence (i.e. concatenated mitochondrial and nuclear DNA). Node numbers refer to divergence time estimations given in Table 2. Thickened branches indicate nodal support by both BA posterior probability (PP) values ≥ 0.95 and ML bootstrap support (BS) ≥ 75. Additional file 1: Table S1 lists support values by node for this phylogeny.

The concatenated mitochondrial and nuclear combined analysis yielded an almost completely resolved tree topology (Figure 7). *Philantomba* is both monophyletic and sister to the remainder of the Cephalophinae. *Cephalophus* was paraphyletic, with *Sylvicapra* as sister to the monophyletic giant duiker clade. The east and west African red duiker lineages are monophyletic and are sister to one another. While their placement is not strongly supported by all methods of estimation, Bayesian support places *C. adersi* as sister to the east and west African red duiker lineages (MP = 61%, ML = 73/65%, BA = 1.0/0.98). Placement of *C. zebra* as sister to the giant and savanna duiker lineages is not supported.

Analyses in BEAST recovered the same topologies as those obtained by BA methods. However, the tree estimated using both nuclear and mitochondrial data was better resolved with higher support and narrower confidence intervals than the tree estimated from nuclear analysis alone. For this reason, we discuss only the results of estimation from both nuclear and mitochondrial data, although ages for nodes recovered in both analyses are presented in Table 2. The split of *Philantomba* from all other members of the Cephalophinae was estimated to have occurred during the late Miocene at 8.73 Ma (6.27-11.43 highest posterior density, HPD). This is followed by the divergence of the giant duiker and *Sylvicapra* lineage from the red duikers at 7.03 Ma (5.02-9.19 HPD), with *C. zebra* and *C. adersi* occupying a sister position relative to these two major groups. This major split is then followed by a subsequent split between the east and west African red duiker lineages at 4.98 Ma (3.58-6.69 HPD) during the Pliocene. With the exception of the dwarf duikers *P. monticola* and *P. maxwelli* all sister duikers species are estimated to have originated during the Pleistocene (< 2.558 Ma). These sister species pairs comprise *C. jentinki* and *C. dorsalis*, *C. nigrifrons* and *C. rufilatus*, *C. natalensis* and *C. harveyi*, *C. spadix* and *C. silvicultor*, and *C. callipygus* and *C. ogilbyi*.

**Table 2:** Divergence times

**Label**	**Node description**	**Total evidence dataset**	**Nuclear dataset**
1	Origin of branch leading to Tragelaphini	17.87 [13.29-22.58]	18.11 [13.79-22.47]
2	Origin of branch leading to *N. moschatus*	15.92 [11.38-20.22]	13.35 [9.27-18.15]
3	Origin of branch leading to *O. oreotragus*	13.73 [9.91-17.72]	
4	Origin of branch leading to *M. kirkii*	13.01 [9.25-16.75]	
5	*Philantomba/Sylvicapra* + *Cephalophus* lineages diverge	8.73 [6.27-11.43]	7.55 [8.49-17.07]
6	Giant and savanna/East and west African red duiker lineages diverge	7.03 [5.02-9.19]	
7	Origin of branch leading to *C. zebra*	6.59 [4.59-8.55]	4.22 [2.50-6.59]
8	Origin of branch leading to *C. adersi*	6.20 [4.37-8.15]	5.25 [4.15-8.66]
9	Giant/savanna duiker lineages diverge	5.87 [4.16-7.78]	
10	East/west African red duiker lineages diverge	4.98 [3.58-6.69]	3.53 [2.19-5.29]
11	*P. monticola/P. maxwelli* divergence	3.93 [2.68-5.31]	3.03 [1.70-4.75]
12	Origin of branch leading to C. niger	3.70 [2.52-4.97]	2.69 [1.59-4.18]
13	*C. spadix* + *C. silvicultor/C. jentinki + C. dorsalis* divergence	3.67 [2.53-4.93]	
14	*T. spekei/T.scriptus* divergence	3.25 [2.15-4.47]	1.91 [0.80-3.44]
15	Origin of branch leading to *C. leucogaster*	3.16 [2.13-4.27]	1.43 [0.58-2.48]
16	*C. jentinki/C. dorsalis* divergence	2.60 [1.74-3.54]	1.63 [0.75-2.80]
17	*C. rufilatus + C. nigrifrons/C. harveyi + C. natalensis* divergence	1.72 [1.18-2.38]	
18	*C. nigrifrons/C. rufilatus* divergence	0.87 [0.51-1.26]	
19	*C. spadix/C. silvicultor* divergence	1.31 [0.80-1.91]	0.98 [0.19-1.29]
20	*C. harveyi*/*C. natalensis* divergence	0.48 [0.25-0.78]	0.49 [0.29-1.52]
21	*C. ogilbyi/C. callipygus* divergence	0.30 [0.74-1.74]	1.03 [0.47-1.82]

Divergence times estimated by BEAST based on either total evidence (mitochondrial and nuclear) or nuclear only datasets. Divergence time estimates are the median age of the posterior distributions in million years of age and the 95% highest posterior density (HPD) intervals are indicated in brackets. Label numbers refer to the topology of Figure 4.

## Discussion

Past attempts to reconstruct the evolutionary history of the Cephalophinae have met with considerable challenge [22-24], likely owing to the recent and rapid radiation of this group [21,22]. Using a combination of mitochondrial and nuclear markers, the present study provides the most well supported phylogeny to date. This study also provides convincing support for the position of *Philantomba* as sister to the remaining Cephalophinae and the recognition of the genus, as recommended by Jansen van Vuuren and Robinson [22]. In contrast, there is no support for *Sylvicapra* whose sister relationship to the giant duikers leaves *Cephalophus* paraphyletic. Instead, our findings suggests that *S. grimmia* represents the sole savanna-dwelling member of the giant duiker lineage within *Cephalophus* and likely evolved from a forest-dwelling common ancestor, further reinforcing Grubb's [28] belief that habitat transitions occur primarily from forest to savannah. While Jansen van Vuuren and Robinson [22] were correct in hypothesizing that the savannah duiker diverged early in the group’s evolutionary history, this study shows that its return to the savannah does not predate the appearance of other forest-dwelling taxa. The present phylogeny also provides much stronger support for the three main lineages of *Cephalophus* identified by Jansen van Vuuren and Robinson [22] and for the first time provides significant support for their placement relative to one another. The failure of this and earlier studies to place *C. adersi* and *C. zebra* in relation to these major lineages is more likely to be a reflection of the rapidity with which these older taxa may have radiated rather than a failure to resolve species nodes.

A comparison of the mitochondrial and nuclear DNA phylogenies also shed light on the evolutionary processes operating within this group. Because mitochondrial DNA has a quarter of the effective population size of nuclear DNA, mitochondrial haplotypes generally sort much more rapidly [29]. Thus, in recently diverged lineages it is expected that the paraphyly observed in mitochondrial DNA should would also be reflected in the nuclear data [30], as is observed for *C. natalensis/C. harveyi* and *C. ogilbyi/C. callipygus*. Incomplete lineage sorting would also explain the paraphyly observed in the nuclear DNA of species that exhibit reciprocally monophyletic relationships in mitochondrial analyses, as appears to be the case for *C. sylvicapra/C. spadix and P. monticola/P. maxwelli*. However, *C. nigrifrons* and *C. rufilatus* do not follow either of these patterns, exhibiting a paraphyletic relationship in mitochondrial analyses and a reciprocally monophyletic relationship in nuclear analyses. One explanation for these findings is that mitochondrial introgression between *C. nigrifrons* and *C. rufilatus,* followed by extensive back-crossing to the original parental taxa, may have obscured mitochondrial relationships but maintained their monophyly at the nuclear level. These two taxa occupy parallel distributions across central African, providing ample opportunities for hybridization. Interestingly, Bayesian analysis of the nuclear data provides support for a sister relationship between *C. nigrifrons* and the *C. natalensis/C. harveyi* clade, indicating that *C. nigrifrons* and *C. rufilatus* may not be sister taxa, as previously mitochondrial analyses suggest [22-24].

We also report the surprising finding that while the origin of most major lineages within the subfamily date to the late Miocene/early Pliocene, many duiker species arose during the Pleistocene. From the mid-Miocene climatic optimum onwards, the earth has experienced a gradual cooling trend that continued through the Plio-Pleistocene [31]. The onset of much drier, colder periods at the boundary between these two epochs and subsequent intensification of glacial cycles throughout the Pleistocene is thought to have provided important opportunities for the diversification and increased turnover of African vertebrate species, including many arid-adapted bovids [21,32]. Grassland expansion during glacial maxima would have confined forest adapted species to fragments of suitable habitat and broken up the formerly contiguous Equatorial African rainforest belt into several major refugia to the west, center and east of Africa [33-35]. Such geographic isolation is thus postulated to have provided ideal opportunities for the allopatric fragmentation and speciation of tropical forest species [36]; reviewed in [9].

Despite the intrinsic appeal of this hypothesis, examples of Pleistocene-era tropical forest speciation are few. Opponents of tropical Pleistocene refuge theory have argued that many species divergence times pre-date the Pleistocene [37] or that forest refugia simply acted as reservoirs of genetic variation but did not drive speciation *per se*[20,38]. Divergence times reported for many central African taxa support this claim [18-20,26,39]; but see [17], although there is ample evidence for intra-specific diversification within many groups including tropical forest mammals [3].

Although our results are surprising, the divergences times we observed here are also consistent with earlier estimates for this group [22]. Moreover, many sister species occupy neighbouring yet allopatrically distributed species ranges. This is witnessed by the east versus west and western central African (i.e. Congo Basin) distribution of *C. spadix* and its sister taxon *C. silvicultor*, a pattern mirrored by the intra-specific structure of the roan antelope (*Hippotragus equinus*) [40]. Similarly, we have also observed splits between taxa that occupy a west versus western central African distribution, notably: 1) *C. jentinki* (west African) from *C. dorsalis* (west and western central African) 2) *C. ogilbyi* (west and western central African) from *C. callipygus* (western central African) and 3) *P. maxwelli* (west Africa) and *P. monticola* (western central, eastern and South African). This pattern is similar to the diversification of the murid rodent (*Praomys misonnei*), a forest associated taxon whose intra-specific distribution is also believed to have been driven by allopatric speciation during refugial isolation [41]. Similarly, there is ample genetic evidence to suggest that the split between eastern and western gorillas (*Gorilla gorilla*) arose during the Pleistocene as a result of climate-induced changes in forest cover this period [13,42]. Lastly, we also observed a pattern of north versus south speciation in the forest-dwelling *C. nigrifrons* and its savanna-dwelling sister species *C. rufilatus*, as well as an east versus south African species split between *C. harveyi* and *C. natalensis*. Remarkably, of these sister species pairs, it is only in the case of *C. ogilbyi* and *C. callipygus* that their ranges overlap. Taken together, these data strongly suggest a pattern of Pleistocene-era fragmentation that led to the distribution of the sister species pairs that we see today.

The divergence times of the duikers however contrasts with findings from another forest bovid subfamily (the Tragelaphini) whose estimated speciation times range across the Miocene and Pliocene between 13 to 3 Ma [26]. More recent studies of *Tragelaphus scriptus* also point to a large sub-specific diversification within this taxon whose timing might also have been driven by Pleistocene climate change [15]. This then raises the question of the nature of species boundaries within the Cephalophinae and other African bovids and whether the timing of the radiation observed here more accurately reflects sub-specific diversification and/or incipient speciation, as is evidenced by several instances of paraphyly and/or hybridization between sister taxa.

Unlike earlier studies of the subfamily, the present study is the first to use both nuclear and mitochondrial data to estimate a species tree, and a fossil calibration point to date divergence times of duikers to the Pleistocene. The pattern of vertebrate radiation observed here fits that advocated by Avise *et al.*[43] where it has been postulated that Pleistocene glaciations either initiated intra-specific differentiation or furthered the speciation of lineages whose origin predated the Pleistocene. Further work should therefore investigate the extent of gene flow between recently derived species and use a coalescent approach to assess divergence times within this group [44].

### Conservation implications

An accurate estimation of a species tree is often a useful precursor for guiding conservation and management decisions [45]. Phylogenetic analysis in this study finds significant support for the recognition of a distinct west African red duiker taxon *C. rubidus* which is geographically-restricted and was previously treated as a subspecies of *C. nigrifrons* within the east African red duiker clade. This apparent conflict lends strength to Jansen van Vuuren & Robinson's [22] recommendation that this taxon should be managed as a distinct species, elevating its conservation status from threatened to endangered [46]. The relationship between *C. callipygus* and the CITES protected species *C. ogilbyi* also appears problematic. Inclusion of nuclear data further substantiates the lack of any clear genetic distinction between these two taxa and is consistent with a history of either recent or on-going hybridization and/or incomplete lineage sorting. Given the results of the present study, it seems unlikely that any mitochondrial or nuclear marker will be able to differentiate these two taxa, posing a challenge to the regulation of the bushmeat trade [24,47] or wildlife monitoring studies of field collected feces [23]. Further work should explore patterns of range-wide population genetic variation between these two taxa in order to better understand their species status and potential for hybridization.

## Conclusions

Fluctuations in climate and increasing aridity over the past few million years are thought to have played an important role in shaping diversification of many African taxa [21,32,48]. Although many previous studies have shown that the majority of speciation events date to the Pliocene (e.g. [18,20,49,50]), Pleistocene-era climatic oscillations are also thought to have played an important role in shaping patterns of diversification, particularly at the population level (e.g. [11,13]). Here we report on a remarkably recent radiation of a group of duiker whose sister species pairs appear to date predominantly to the Pleistocene. As is the case for other forest artiodactyls, taxa within this group are tied to forest environments, thus highlighting the potential importance that Pleistocene refugia may have played in the speciation of forest-dwelling species. Data from this study also highlight several areas of inconsistency between our current understanding of duiker taxonomy and the evolutionary relationships depicted here. Consistent with their recent origin, several sister species groups exhibit paraphyletic relationships and/or evidence of recent hybridization. Further work should therefore aim to sample more widely across these sister taxa in order to better understand the geographic range of paraphyletic lineages and identify potential areas of introgression. These findings may also prove particularly relevant to future conservation efforts, given that many species are presently regulated under the Convention for Trade in Endangered Species and are therefore targets for the bushmeat trade.

## Methods

Tissue was sampled from 24 individuals within the Cephalophinae, representing all eighteen species recognized by the International Union for Conservation of Nature (IUCN) [51]. Sequences were also obtained from Genbank of the newly discovered species, *P. walteri*[52]*,* and one taxon that is considered a subspecies by IUCN (*C. rubidus*) (Additional file 2). With respect to a suitable outgroup, recent mitochondrial studies have suggested that the klipspringer (*Oreotragus oreotragus*) may be sister to the Cephalophinae [53,54]. However, nuclear markers [25] and supertree analysis [55] do not provide support for this relationship, or for any consistent sister group to the Cephalophinae. Given the uncertainty of these relationships, we have included not only *O. oreotragus* as a candidate outgroup but also two other closely related taxa within the subfamily Antelopinae (the suni *Neotragus moschatus* and Kirk's dik-dik *Madoqua kirkii*) alongside two more divergent species within the subfamily Bovinae, the bushbuck (*Tragelaphus scriptus*) and the sitatunga (*T. spekei*).

Samples were obtained from bushmeat market surveys conducted in collaboration with the Wildlife Conservation Society (WCS) in Gabon, or donated by zoos and other researchers. With the exception of the easily distinguishable *P. monticola* and *T. spekei*, a photographic record was used to verify the species identity of all WCS collected bushmeat samples. Tissue samples of several species obtained from the San Diego Zoo and a fecal sample taken from *C. jentinki* at Gladys Porter Zoo were accompanied by species records. Details for all remaining samples are found in [22-25,56].

DNA from all bushmeat and some San Diego Zoo tissues was extracted using a standard phenol-chloroform extraction method [57]. DNA provided by Jansen van Vuuren was extracted according to the methods described in [22]. Other samples provided by the San Diego Zoo were obtained as genomic DNA extracts. The *C. jentinki* fecal sample was extracted using the QIAamp DNA Stool Minikit (Qiagen) in a designated room and a blank was included to control for DNA contamination. The *C. harveyi* sample AB05 was extracted from blood using a salt-based extraction method [58].

Portions of two coding mDNA genes were included in phylogenetic analyses: 514 bp of the cytochrome *b* (cyt*b*) gene and 658 bp of the cytochrome *c* oxidase subunit 1 (COX1). See Additional file 2 for GenBank accession numbers for sequences obtained from previous studies or from the current study. Most (n = 36) of the cyt*b* sequences were previously published [22,23,52,59]. All Genbank sequences were trimmed to match the cyt*b* region employed by Ntie *et al.*[23]. The cyt*b* gene fragment from *C. jentinki* was amplified according to published primers and protocols [23]. Similarly, most (n = 31) of the COX1 sequences were previously published [24,52] and an additional five samples were amplified according to published protocols [24]. To test for the potential presence of non-functional nuclear translocated copies of mitochondrial DNA (Numts), each of the mitochondrial gene sequences were translated to amino acids in the program MEGA v3. [60]. No evidence of frameshifts or stop codons were found.

Four nuclear DNA markers were also amplified and sequenced using published primers and PCR conditions [61]. These markers span introns within four genes: stem cell factor (MGF), protein-kinase-CI (PRKCl), B-spectrin non-erythrocytic (SPTBN1) and thyrotropin (THY). Internal primers were designed and used to amplify smaller fragments for samples that were highly degraded or difficult to amplify (Additional file 3). PRKCl, SPTBN1 and THY sequences for outgroup taxa *M. kirkii, N. moschatus,* and *O. oreotragus* were obtained from Genbank [25]. Following amplification, all PCR products were purified using ExoAp [62] and then sequenced on both strands using the BigDye Terminator Cycle Sequencing Kit v1.1 (ABI). Resulting products were run on a 3100 ABI automated DNA sequencer. Forward and reverse sequences were edited using the program SEQUENCHER v4.1.1 (Gene Codes Corporation, Ann Arbor, MI, USA). For nuclear loci, heterozygous individuals were verified by the presence of two similarly sized, overlapping peaks observed in both sequencing directions, and were coded using standard IUPAC ambiguity codes.

The incongruence length difference test (ILD; [63]) implemented in PAUP* vers. 4.0b10 [64] was used to evaluate incongruence between mitochondrial genes, nuclear introns and between combined mitochondrial and nuclear datasets. These ILD tests used 1,000 randomized partitions of the data and a heuristic search on each randomization to obtain the sum of tree lengths for each partition. The models of nucleotide substitution that best fit the data were selected by jModelTest [65,66] under the Bayesian information criterion (BIC; [67]).

We also tested for topological concordance between phylogenetic trees derived from mitochondrial and nuclear DNA data partitions using the likelihood-based SH test [68] implemented in PAUP*. The ML tree for mitochondrial dataset was first estimated using jModeltest parameters for that partition. A second ML search on the same dataset was then carried out using the nuclear topology as a constraint. The significance of the difference in the sum of the site-wise log likelihoods of the two trees (unconstrained versus constrained) was then assessed using the SH test. A reciprocal test of topology was also conducted by first estimating the ML tree from the concatenated nuclear dataset and then estimating a constrained ML tree forced to fit the mitochondrial topology.

Gene trees were estimated for each of the four nuclear introns and the combined mitochondrial genes using maximum parsimony (MP), maximum likelihood (ML), and Bayesian (BA) methods (see below). Additionally, nuclear introns were concatenated with and without mitochondrial sequences into a single data matrix for species tree estimation using MP, ML, and BA methods. Nuclear sequences were not available for *C. weynsi*, *C. rubidus* and *P. walteri* either because the sample failed to amplify or because no tissue was available for the present study.

All MP analysis were performed in PAUP*. For each analysis, preliminary maximum parsimony searches were conducted using heuristic search methods with tree bisection reconnection (TBR) branch swapping, collapse of zero-length branches, all characters weighted equally, and 100 replicates of the random addition starting tree option. A nonparametric bootstrap test [69] was carried out using 300 replicates. The “Max Trees” was set to 50,000 for both initial searches and for the bootstrap tests.

Maximum likelihood analyses using a single model of nucleotide substitution for individual introns and concatenated mitochondrial and nuclear matrices were performed in PAUP* vers. 4.0b10 for UNIX. Heuristic searches were carried out using the TBR branch swapping algorithm, collapsing zero-length branches and using 100 replicates of the random addition option for the starting tree. Nonparametric bootstrap values were calculated from a consensus of the 300 replicate searches.

Two additional ML searches were conducted in RAxML vers. 7.0.4 [70] in which each nuclear intron was assigned its own model of nucleotide substitution with or without the inclusion of the mitochondrial data as an additional partition. Within each heuristic search, 500 discrete starting trees were used and a bootstrap consensus tree was estimated from the resulting trees. Each search used a GTR model of nucleotide substitution with the gamma model of rate heterogeneity initiated from a complete random starting tree. Model parameters were optimized to a likelihood difference of 0.00001. Each bootstrap analysis was repeated twenty times to explore tree space and ensure that each analysis converged on a similar likelihood score.

Bayesian analyses were carried out using the Metropolis-coupled Markov chain Monte Carlo (MCMC) methods implemented in MrBayes vers. 3.1.2 [71]. Each analysis included two independent, simultaneous runs. Each run consisted of four chains, one of which was the ‘cold’ chain and three of which were the chains heated according to the default heating method parameters of MrBayes. Each chain was run for up to 50 million generations, initiated from a random starting tree. The chain was sampled every 1,000 generations for a total of up to 50,001 tree samples per run. As simultaneous runs converged onto the stationary distribution, the average standard deviation of split frequencies should approach zero. Therefore, convergence was determined when the standard deviation of split frequencies between simultaneous runs was less than 0.01, as calculated by MrBayes. Additionally, trace files were evaluated with the program Tracer vers. 1.5 [72] and 10% of points collected prior to chain stationarity were discarded as burn-in. The parameter and tree samples from the two simultaneous runs were combined and summarized using the sump and sumt commands, respectively. For the first set of runs, BA searches assumed a single model of nucleotide substitution across the dataset. A second analysis was carried out in which nuclear genes were partitioned to allow each gene to have its own model of nucleotide substitution. This analysis was repeated with the mitochondrial DNA included as an additional partition. Bayes Factor (BF) analysis was used to investigate the effects of partitioning on the Bayesian analysis. Following [73], two times the natural logarithm of the Bayes Factor was calculated as 2 ln BF(21) = 2[ln(hm2)-ln(hm1)]; where hm2 and hm1 are the harmonic means of the post-burn-in likelihood values for the partitioned and un-partitioned analyses, respectively as estimated using the sump command in Mr. Bayes. The threshold of 2 ln BF > 10 was taken as strong evidence for the partitioned model [74]. Although the harmonic mean is not the best estimator of the marginal likelihoods used to compute the Bayes Factor, alternative methods [75-77] are either computationally intensive or not readily implementable at this time.

Divergence times and tree topology were simultaneously estimated using the program BEAST vers. 1.6.1 [78]. BEAST analyses were run with and without the mitochondrial data because ILD tests indicate conflicting signal between nuclear and mitochondrial genomes. The likelihood ratio test implemented in PAUP* was used to determine if a molecular clock hypothesis could be rejected for each locus. Radiometrically dated fossil remains suggest that the earliest appearance of the Cephalophinae was between 6.31 – 5.65 Ma [21], which coincides well with the estimated oldest speciation event within Cephalophinae at 5.3 Ma (± 53,434 years) [22], using a cyt*b* molecular clock calibration for the family Bovidae [59]. From this information, the prior on the age of the node uniting all taxa within the Cephalophinae was set as a lognormal distribution with an upper bound set as an offset value of 5.3 Ma, a log mean of 0.32 Ma from this offset value and log standard deviation of 1 Ma such that 95% of the prior probability encompassed the timeframe suggested by fossil evidence. The prior on the stem of the tree was set as a normal distribution with a mean of 20.1 Ma and a SD of 2.25 Ma. This prior distribution encompasses the dates (16.4 – 23.8 Ma) within which the split between the Bovinae and Antilopinae is believed to have occurred [53]. We unlinked the substitution models across nuclear genes, but left the mitochondrial genes linked. Because a molecular clock hypothesis could be rejected for all loci (MGF: *χ*^2^ = 150.96398, PRKCl: *χ*^2^ = 60.019, STBN1: *χ*^2^ = 115.41636, THY: *χ*^2^ = 128.10964, mitochondrial: *χ*^2^ = 223.16424, d.f. = 30, p <0.05), we used a relaxed, uncorrelated lognormal clock model and a Yule tree prior as implemented by the program. All other priors were left at their default settings. Two independent MCMC chains were run for 10 million generations and sampled every 1000 states, after which convergence was determined when the combined independent chains yielded posterior probability effective sample sizes (ESS) greater than 200. After examining trace files, the first 25% of the samples were discarded as burn-in and the remaining 7,501 samples from each run were combined in Logcombiner for a total of 15,002 sample genealogies per analysis. Tree Annotator was used to summarize the trees into a single maximum clade credibility tree.

## Abbreviations

CI, Consistency index; RI, Retention index; MP, Maximum parsimony; ML, Maximum likelihood; BA, Bayesian analysis; BS, Bootstrap support; PP, Posterior probability; WCS, Wildlife conservation Society; cytb, Cytochrome b; COX1, Cytochrome c oxidase subunit 1; MGF, Stem cell factor; PRKCl, Protein-kinase-CI; SPTBN1, B-spectrin non-erythrocytic; THY, Thyrotropin; ILD, Incongruence length difference test; BIC, Bayesian information criterion; SH, Shimodaira-hasegawa; TBR, Tree bisection reconnection; MCMC, Markov chain monte carlo; BF, Bayes factor.

## Authors' contributions

ARJ conceived of the study and its design, carried out extractions, primer modifications, DNA sequencing, alignment, and analyses and drafted the manuscript. NMA helped in all of these aspects of the study. Both authors read and approved the final manuscript.

## Supplementary Material

Additional file 1 **Support values.** Excel sheet containing posterior probabilities (PP) or bootstrap support (BS) for the nodes in Figures 1, 2, 3 and 4. Values in column A are BEAST PP. Values in columns B are un-partitioned BA PP/partitioned BA PP. Values in columns C are un-partitioned ML BS/partitioned ML BS (MP BS). Values in columns D are BA PP/ML BS/MP BS. Values in bold are PP ≥ 0.95 or BS ≥ 75. Click here for file

Additional file 2 **Sample information.** Species, sample ID, country of origin where known, material donor, and GenBank accession numbers of all samples and sequences used in the present study. Highlighting of accession number corresponds to initial publication as follows: pink [23]; blue [24]; orange [52]; white [22]; red [59]; yellow [61]; green in present study. Click here for file

Additional file 3 **Primers.** Internal primers designed to work in combination with the primers and protocols designed by Matthee et al. [61] (indicated by an asterisk) to amplify overlapping fragments for degraded samples, using a modified [Mg^2+^ and annealing temperature. (XLSX 12 kb) Click here for file

## References

[B1] Haffer J Alternative models of vertebrate speciation in Amazonia: an overview Biodivers Conserv 1997 6 451 476

[B2] Da Silva M Patton J Molecular phylogeography and the evolution and conservation of Amazonian mammals Mol Ecol 1998 7 475 486 9628001 10.1046/j.1365-294x.1998.00276.x

[B3] Hewitt G The structure of biodiversity - insights from molecular phylogeography Front Zool 2004 1 4 15679920 10.1186/1742-9994-1-4 PMC544936

[B4] Haffer J Speciation in Amazonian forest birds Sci New York 1969 165 131 137 10.1126/science.165.3889.131 17834730

[B5] Colinvaux P De Oliveira P Bush M Amazonian and neotropical plant communities on glacial time-scales: the failure of the aridity and refuge hypotheses Quat Sci Rev 2000 19 141 169

[B6] Willis K Paleocology: the refugial debate Sci 2000 287 1406 1407 10.1126/science.287.5457.1406 10722388

[B7] Lessa E Cook J Patton J Genetic footprints of demographic expansion in North America, but not Amazonia, during the Late Quaternary Proc Natl Acad Sci USA 2003 100 10331 10334 12913123 10.1073/pnas.1730921100 PMC193561

[B8] Querouil S Verheyen E Dillen M Colyn M Patterns of diversification in two African forest shrews: Sylvisorex johnstoni and Sylvisorex ollula (Soricidae, Insectivora) in relation to paleo-environmental changes Mol Phylogenet Evol 2003 28 24 37 12801469 10.1016/s1055-7903(03)00027-7

[B9] Plana V Mechanisms and tempo of evolution in the African Guineo-Congolian rainforest. Philos Trans R Soc Lond Ser B Biol Sci 2004 359 1585 1594 10.1098/rstb.2004.1535 PMC1693432 15519974

[B10] Bowie R Fjeldså J Hackett S Bates J Crowe T Coalescent models reveal the relative roles of ancestral polymorphism, vicariance, and dispersal in shaping phylogeographical structure of an African montane forest robin Mol Phylogenet Evolution 2006 38 171 188 10.1016/j.ympev.2005.06.001 16024259

[B11] Born C Insights into the biogeographical history of the Lower Guinea Forest Domain: evidence for the role of refugia in the intraspecific differentiation of Aucoumea klaineana Mol Ecol 2011 20 131 142 21091559 10.1111/j.1365-294X.2010.04919.x

[B12] Nicolas V The roles of rivers and Pleistocene refugia in shaping genetic diversity in Praomys misonnei in tropical Africa J Biogeogr 2011 38 191 207

[B13] Anthony NM The role of Pleistocene refugia and rivers in shaping gorilla genetic diversity in central Africa Proc Natl Acad Sci USA 2007 104 20432 20436 18077351 10.1073/pnas.0704816105 PMC2154448

[B14] Trauth M Larrasoana J Mudelsee M Trends, rhythms and events in Plio-Pleistocene African climate Quat Sci Rev 2009 28 399 411

[B15] Moodley Y Bruford M Molecular biogeography: towards an integrated framework for conserving pan-African biodiversity PLoS One 2007 2 454 10.1371/journal.pone.0000454 PMC1866246 17520013

[B16] Brown D Extensive population genetic structure in the giraffe BMC Biol 2007 5 57 18154651 10.1186/1741-7007-5-57 PMC2254591

[B17] Janssens S Fischer E Stevart T New insights into the origin of two new epiphytic Impatiens species (Balsaminaceae) from West Central Africa based on molecular phylogenetic analyses Taxon 2010 59 1508 1518

[B18] Couvreur T Chatrou L Sosef M Richardson J Molecular phylogenetics reveal multiple tertiary vicariance origins of the African rain forest trees BMC Biol 2008 6 54 19087283 10.1186/1741-7007-6-54 PMC2628871

[B19] Holstein N Renner S A dated phylogeny and collection records reveal repeated biome shifts in the African genus Coccinia (Cucurbitaceae) BMC Evol Biol 2011 11 28 21269492 10.1186/1471-2148-11-28 PMC3041684

[B20] Tolley K Ancient forest fragmentation or recent radiation? Testing refugial speciation models in chameleons within an African biodiversity hotspot J Biogeogr 2011 38 1748 1760

[B21] Vrba E Vrba E, Denton G, Partridge T, Burckle L The fossil record of African antelopes (Mammalia, Bovidae) in relation to human evolution and paleoclimate Paleoclimate and evolution, with emphasis on human origins 1995 Yale University Press, New Haven 385 424

[B22] Jansen van Vuuren Vuuren B Robinson T Retrieval of four adaptive lineages in duiker antelope: evidence from mitochondrial DNA sequences and fluorescence in situ hybridization Mol Phylogenet Evol 2001 20 409 425 11527467 10.1006/mpev.2001.0962

[B23] Ntie S A molecular diagnostic for identifying central African forest artiodactyls from faecal pellets Anim Conserv 2010 13 80 93

[B24] Johnston A Morikawa M Ntie S Anthony N Evaluating DNA barcoding criteria using African duiker antelope (Cephalophinae) as a test case Conserv Genet 2011 12 1173 1182

[B25] Matthee C Davis S Molecular insights into the evolution of the family Bovidae: a nuclear DNA perspective Mol Biol Evol 2001 18 1220 1230 11420362 10.1093/oxfordjournals.molbev.a003908

[B26] Willows-Munro S Robinson T Matthee C Utility of nuclear DNA intron markers at lower taxonomic levels: phylogenetic resolution among nine Tragelaphus spp Mol Phylogenet Evol 2005 35 624 636 15878131 10.1016/j.ympev.2005.01.018

[B27] Drummond A Ho S Phillips M Rambaut A Relaxed phylogenetics and dating with confidence Plos Biol 2006 4 699 710 10.1371/journal.pbio.0040088 PMC1395354 16683862

[B28] Grubb P Patterns of speciation in African Mammals Bull Carnegie Mus Nat Hist 1978 6 152 165

[B29] Funk D Omland K Species-level paraphyly and polyphyly: frequency, causes, and consequences, with insights from animal mitochondrial DNA Annu Rev Ecol Evol Syst 2003 34 397 423

[B30] Zink R Barrowclough G Mitochondrial DNA under siege in avian phylogeography Mol Ecol 2008 17 2107 2121 18397219 10.1111/j.1365-294X.2008.03737.x

[B31] Zachos J Pagani M Sloan L Thomas E Billups K Trends, rhythms and aberrations in global climate 65 Ma to present Sci 2001 292 686 693 10.1126/science.1059412 11326091

[B32] deMenocal P Plio-Pleistocene African Climate Sci 1995 270 53 59 10.1126/science.270.5233.53 7569951

[B33] van Zinderen Bakker E Mercer H Major late cainozoic climate events and paleoenvironmental changes in Africa viewed in a worldwide context Paleogeogr Paleoclimatol Paleoecol 1986 56 217 235

[B34] Hamilton A Taylor D History of climate and forests in tropical Africa during the last 8 million years Clim Chang 1991 19 65 78

[B35] Maley J The African rainforest – main characteristics of changes in vegetation and climate from the upper cretaceous to the quaternary Proc R Soc Edinb 1996 104N 31 73

[B36] Prance G A review of the phytogeographic evidence for Pleistocene climate change in the neotropics Ann Mo Bot Gar 1982 69 594 624

[B37] Moritz C Patton J Schneider C Smith T Diversification of rainforest faunas: an integrated molecular approach Annu Rev Ecol Syst 2000 31 533 563

[B38] Fjelds J Geographical patterns for relict and young species of birds in Africa and South America and implications for conservation priorities Biodivers Conserv 1994 3 207 226

[B39] Aduse-Poku K Vingerhoedt E Wahlberg N Out-of-Africa again: a phylogenetic hypothesis of the genus Charaxes (Lepidoptera: Nymphalidae) based on five gene regions Mol Phylogenet Evol 2009 53 463 478 19580878 10.1016/j.ympev.2009.06.021

[B40] Alpers D Jansen van Vuuren B Arctander P Population genetics of the roan antelope (Hippotragus equinus) with suggestions for conservation Mol Ecol 2004 13 1771 1784 15189202 10.1111/j.1365-294X.2004.02204.x

[B41] Nicolas V Missoup A Denys C The roles of rivers and Pleistocene refugia in shaping genetic diversity in Praomys misonnei in tropical Africa J Biogeogr 2011 38 191 207

[B42] Thalmann O Fischer A Lankester F Pääbo S Vigilant L The complex evolutionary history of gorillas: insights from genomic DNA Mol Biol Evol 2007 24 146 158 17065595 10.1093/molbev/msl160

[B43] Avise J Walker D Johns G Speciation durations and Pleistocene effects on vertebrate phylogeography Proc R Soc Lond Ser B-Biol Sci 1998 265 1707 1712 10.1098/rspb.1998.0492 PMC1689361 9787467

[B44] Hey J Isolation with migration models for more than two populations Mol Biol Evol 2010 27 905 920 19955477 10.1093/molbev/msp296 PMC2877539

[B45] Crandall K Bininda-Emonds O Mace G Wayne R Considering evolutionary processes in conservation biology Trends Ecol Evol 2000 15 290 295 10856956 10.1016/s0169-5347(00)01876-0

[B46] Kingdon J The Kingdon Field Guide to African Mammals 1997 New York and London, Academic Press

[B47] Eaton M Barcoding bushmeat: molecular identification of Central African and South American harvested vertebrates Conserv Genet 2009 11 1389 1404

[B48] Hamilton A Taylor D History of climate and forests in tropical Africa during the last 8 million years Clim Change 1991 19 65 78

[B49] Damm S Dijkstra K Hadrys H Red drifters and dark residents: the phylogeny and ecology of a Plio-Pleistocene dragonfly radiation reflects Africa’s changing environment (Odonata, Libellulidae, Trithemis) Mol Phylogenet Evol 2010 54 870 82 20004729 10.1016/j.ympev.2009.12.006

[B50] Voelker G Outlaw R Bowie R Pliocene forest dynamics as a primary driver of African bird speciation Global Ecol and Biogeogr 2010 19 111 121

[B51] IUCN 2011. IUCN Red List of Threatened Species. Version 2011.2, http://www.iucnredlist.org. Downloaded on 5 August 2011

[B52] Colyn M Discovery of a new duiker species (Bovidae: Cephalophinae) from the Dahomey Gap West Africa Zootaxa 2010 2637 1 30

[B53] Hassanin A Douzery E The tribal radiation of the family Bovidae (Artiodactyla) and the evolution of the mitochondrial cytochrome b gene Mol Phylogenet Evol 1999 13 227 243 10603253 10.1006/mpev.1999.0619

[B54] Agnarsson I May-Collado L The phylogeny of Cetartiodactyla: the importance of dense taxon sampling, missing data, and the remarkable promise of cytochrome b to provide reliable species-level phylogenies Mol Phylogenet Evol 2008 48 964 985 18590827 10.1016/j.ympev.2008.05.046

[B55] Hernandez Fernandez M Vrba E A complete estimate of the phylogenetic relationships in Ruminantia: a dated species-level supertree of the extant ruminants Biol Rev (Cambridge) 2005 80 269 302 10.1017/s1464793104006670 15921052

[B56] Bowkett A Rovero F Marshall A The use of camera-trap data to model habitat use by antelope species in the Udzungwa mountain forests, Tanzania Afr J Ecol 2008 46 479 487

[B57] Sambrook J Russell D Molecular cloning: A Laboratory Manual 2001 Cold Springs Harbor Laboratory Press, Cold Springs Harbor

[B58] Aljanabi S Martinez I Universal and rapid salt-extraction of high quality genomic DNA for PCR-based techniques Nucleic Acid Res 1997 25 4692 4693 9358185 10.1093/nar/25.22.4692 PMC147078

[B59] Matthee C Robinson T Cytochrome b phylogeny of the family bovidae: Resolution within the Alcelaphini, Antilopini, Neotragini, and Tragelaphini Mol Phylogenet Evol 1999 12 31 46 10222159 10.1006/mpev.1998.0573

[B60] Kumar S Tamura K Nei M MEGA: integrated software for molecular evolutionary genetics analysis and sequence alignment Briefings Bioinform 2004 5 150 163 10.1093/bib/5.2.150 15260895

[B61] Matthee C Burzlaff J Taylor J Davis S Mining the mammalian genome for artiodactyl systematics Syst Biol 2001 50 367 390 12116581

[B62] Glenn T Schable N Isolating microsatellite DNA loci Molecular Evolution: Producing the Biochemical Data, Part B 2005 Elsevier Academic Press Inc, San Diego

[B63] Farris J Constrcting a significance test for incongruence Syst Biol 1995 44 570 572

[B64] Swofford D PAUP*: phylogenetics analysis using parsimony (*and other mothods). version 4.0.b10 2002 Sinauer Associates, Sunderland, MA

[B65] Guindon S Gascuel O A simple, fast, and accurate algorithm to estimate large phylogenies by maximum likelihood Syst Biol 2003 52 696 704 14530136 10.1080/10635150390235520

[B66] Posada D jModelTest: phylogenetic model averaging Mol Biol Evol 2008 25 1253 1256 18397919 10.1093/molbev/msn083

[B67] Schwarz G Estimating dimensions of a model Ann Stat 1978 6 461 464

[B68] Shimodaira H Hasegawa M Multiple comparisons of log likelihoods with applications to phylogenetic inference Mol Biol Evol 1999 16 1114 1116

[B69] Felsenstein J Confidence-limits on phylogenies - an approach using the bootstrap Evol 1985 39 783 791 10.1111/j.1558-5646.1985.tb00420.x 28561359

[B70] Stamatakis A RAxML-VI-HPC: maximum likelihood-based phylogenetic analyses with thousands of taxa and mixed models Bioinforma 2006 22 2688 2690 10.1093/bioinformatics/btl446 16928733

[B71] Ronquist F Huelsenbeck J MrBayes 3: Bayesian phylogenetic inference under mixed models Bioinforma 2003 19 1572 1574 10.1093/bioinformatics/btg180 12912839

[B72] Rambaut A Drummond A Tracer 2007 version 1.4, Available from http://beast.bio.ed.ac.uk/Tracer

[B73] Brandley M Schmitz A Reeder T Partitioned Bayesian analyses, partition choice, and the phylogenetic relationships of scincid lizards Syst Biol 2005 54 373 390 16012105 10.1080/10635150590946808

[B74] Kass R Raftery A Bayes factors J Am Stat Assoc 1995 90 773

[B75] Lartillot N Philippe H Computing Bayes factors using thermodynamic integration Syst Biol 2006 55 195 207 16522570 10.1080/10635150500433722

[B76] Fan Y Wu R Chen M Kuo L Lewis P Chossing among partition models in Bayesian phylogenetics Mol Biol Evol 2010 28 523 532 20801907 10.1093/molbev/msq224 PMC3002242

[B77] Xie W Lewis P Fan Y Kuo L Chen M Improving marginal likelihood estimation for Bayesian phylogenetic model selection Syst Biol 2011 60 150 160 21187451 10.1093/sysbio/syq085 PMC3038348

[B78] Drummond A Rambaut A BEAST: Bayesian evolutionary analysis by sampling trees Bmc Evol Biol 2007 7 214 17996036 10.1186/1471-2148-7-214 PMC2247476

